# Ru-Doped Wells–Dawson Polyoxometalate as Efficient Catalyst for Glycerol Hydrogenolysis to Propanediols

**DOI:** 10.3390/ma12132175

**Published:** 2019-07-06

**Authors:** Amalie Modvig, Chiraphat Kumpidet, Anders Riisager, Jakob Albert

**Affiliations:** 1 Centre for Catalysis and Sustainable Chemistry, Department of Chemistry, Technical University of Denmark, DK-2800 Kgs. Lyngby, Denmark; 2 Lehrstuhl für Chemische Reaktionstechnik, Friedrich-Alexander-Universität Erlangen-Nürnberg, Egerlandstr. 3, 91058 Erlangen, Germany

**Keywords:** Wells–Dawson polyoxometalate, ruthenium, characterization, hydrogenolysis, glycerol

## Abstract

A Ru-doped phospho-tungstic Wells–Dawson polyoxometalate (POM) was successfully applied as homogeneous catalyst for glycerol hydrogenolysis in aqueous media. The synthesized compound showed superior catalytic activity compared to classical homogeneous/heterogeneous Ru catalysts like RuCl_3_ and Ru/C under identical reaction conditions, whereas the analogous POM doped with Pd or Pt proved far less activity. Detailed characterization of the POMs was performed using ^31^P-NMR to identify characteristic phosphorous peaks of the heteroatoms, infrared spectroscopy (ATR-FTIR) to confirm characteristic P-O and W-O-W vibrations, powder XRD for comparison of crystal structures, and X-ray fluorescence (XRF) and inductive-coupled plasma (ICP) analysis to determine elemental composition. Variation of the reaction parameters for the best performing Ru-doped POM catalyst showed that substrate concentration played an important role for both product selectivity and conversion. Moreover, medium hydrogen pressure and high stirring speed were key factors to obtain highly selective conversion of glycerol to 1,2-propanediol.

## 1. Introduction

Currently, industrial production of chemicals is almost entirely based on fossil feedstock. However, demands for the development of more sustainable processes have promoted development of processes based on alternative renewable feedstocks. Biomass offers a promising renewable feedstock with a potential to improve the overall economics and sustainability of the production of bulk chemicals [1,2]. The market for biomass-derived chemicals is growing and predicted to increase to 22% of the market share in 2025 [3,4].

Glycerol (GL) is a major byproduct of biodiesel manufacturing produced in an amount corresponding to around 10 wt.% of the biodiesel production [5,6]. The production of biodiesel is expected to increase to 36.9 million metric tons in 2020, thereby increasing the availability of bio-GL [7]. Several catalytic transformations of GL into value-added chemicals have been reported including steam reforming, oxidation, dehydration, acetylation, esterification, etherification, carboxylation, and chlorination [8,9,10]. Nevertheless, hydrogenolysis of GL into propanediols (PDs) is one of the most attractive approaches for GL valorization due to the wide applicability of PDs on a large scale [11,12].

1,2-Propanediol (1,2-PD), or propylene glycol, is an important chemical extensively used as a monomer for polyester resin. Other industrial applications are found in the food, pharmaceutical, cosmetic, and animal feed industries. The annual production of 1,2-PD in the US was estimated to 635 thousand metric tons in 2001, with an increasing consumption rate of 4% [13]. Conventional 1,2-PD is produced by hydration of propylene oxide derived from propylene, whereas bio-1,2-PD production is based on carbohydrate conversion, such as sorbitol or xylitol, *via* hydrogenolysis under high temperatures and pressures with a metal catalyst [14,15,16]. Selective catalytic hydrogenolysis of GL provides an attractive, greener alternative to the current fossil-based manufacturing process of 1,2-PD, as the latter involves several hydrogenation and hydration steps to stabilize the reactive intermediates, resulting in low selectivity and insufficient product purity [5,6,7,13]. Here, we have examined the selective hydrogenolysis of GL into 1,2-PD using polyoxometalate-based catalysts.

**Figure 1:**
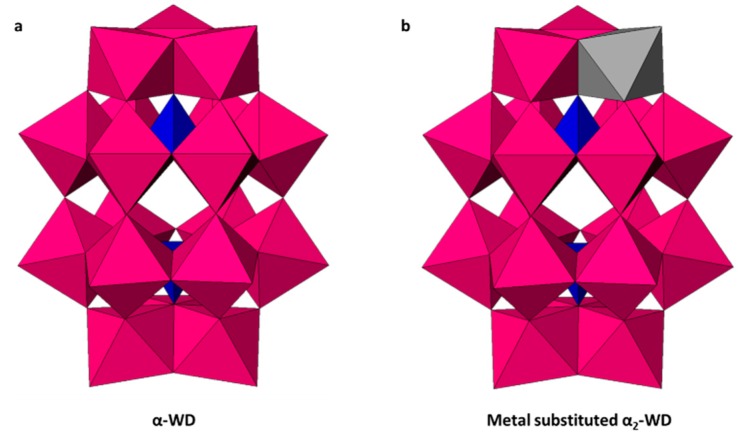
Illustrations of (**a**) the α-Wells–Dawson polyoxometalate (POM) [P_2_W_18_O_62_]^6−^ (α-WD) and (**b**) the metal-substituted α_2_-Wells–Dawson (WD) species with the substituted metal shown in grey, MO_6_, in pink and XO_4_ in blue.

Polyoxometalates (POMs) are inorganic polyatomic cluster ions, which are often assembled around a central heteroatom. The discrete ionic structure provides different acidic and redox properties than other common metal-oxides used in catalysis, e.g., zeolites and metal-oxide supports [17]. Wells–Dawson (WD) POMs have the general formula [X_2_M_18_O_62_]^n−^ and consist of two trilacunary Keggin structures (XM_9_^n−^) linked in a corner-sharing fashion (Figure 1a). Removal of a metal oxide (e.g., tungstate oxide) unit leaves a free position for incorporation of a catalytic active metal, as illustrated in Figure 1b. Phospho-tungstic WD POMs are highly stable in both solid-state, aqueous solutions below pH 6 as well as in some organic solvents [18,19]. Their remarkable redox properties have found application in oxidation of organic compounds, hydrogenation of cyclohexene, and Michael additions of alcohols [20,21,22,23,24]. 

Hydrogenolysis of GL can be regarded as a two-step process involving acid-catalyzed GL dehydration affording a double bond, which is then selectively hydrogenated to yield the desired PD [6,7]. The acidity of the applied catalyst affects the position of remaining hydroxyl groups on PD [6,7]. The WD POM provides a strong acid source, and tuning of the chemical properties of the material by selective substitution of an addenda atom provides a possible active site for hydrogenolysis. The remarkable redox properties and high thermal stability of POMs have been reported to be preserved after substitution of an addenda atom, affording promising replacements for metalloporphyrins in redox and electrochemical reactions [25,26]. 

In this work, we report the selective hydrogenolysis of glycerol into 1,2-PD in aqueous solution using a Ru, Pt, and Pd metal-doped α_2_-WD POMs. Effective hydrogenations have previously been reported using Ru-, Pt-, and Pd-complexes as catalysts [27,28,29]. Tungsten was selected as the addenda atom of the POM due to its high stability and ability to promote efficient hydrogenolysis reactions [27].

## 2. Materials and Methods 

### 2.1. Chemicals 

Reagents were of analytical grade and used without further purification: Sodium tungstate dihydrate (Na_2_WO_4_·2H_2_O, ≥99.0%), hydrochloric acid (HCl, 37 wt.%), phosphoric acid (H_3_PO_4_, 85 wt.%), potassium chloride (KCl, ≥99.0%), potassium hydrogencarbonate (KHCO_3_, ≥99.5%), ruthenium(III) chloride hydrate (RuCl_3_·xH_2_O, ≥99.0%), palladium(II) chloride (PdCl_2_, 99%), and platinum(II) chloride (PtCl_2_, 98%) were all purchased from Sigma Aldrich. The substrate glycerol (double distilled, 99.5%) was purchased from VWR. The heterogeneous catalysts with 5 wt.% metal on activated carbon (Ru/C, 5 wt.% Ru; Pd/C, 5 wt.% Pd; Pt/C, 5 wt.% Pt) were purchased from Alfa Aesar. Moreover, high-purity-grade hydrogen 5.0 and nitrogen 4.6 gases were obtained from Linde AG.

### 2.2. Synthesis of WD-POMs

The metal-doped α_2_-WD POMs were synthesized by a three-step procedure as described below. Synthesis of α-WD (α-K_6_P_2_W_18_O_62_—Step 1): Isomeric pure α-phospho-tungstic-WD (α-WD) POM was synthesized and precipitated as the potassium salt α-K_6_P_2_W_18_O_62_ according to a literature procedure by Nadjo et al. and modified by Graham and Finke [30,31]. Deionized water in the synthesis was replaced by millipore water. Yield 36.2 g (76%). ^31^P-NMR (162 MHz, D_2_O) δ/ppm: −12.78 (2.00; α-WD).

Synthesis of α_2_-WD (α_2_-K_10_P_2_W_17_O_61_—Step 2): The monolacunary α_2_-phospho-tungstic-WD POM was synthesized as the potassium salt according to literature procedure by Finke [32]. Millipore water was used in place of deionized water. Yield 5.9 g (85%). ^31^P-NMR (162 MHz, D_2_O) δ/ppm: 5.06 (0.09; impurity), −6.97 (0.79; α_2_-WD), −7.72 (0.03; impurity), −13.71 (−0.07; impurity), −14.13 (2.00; α_2_-WD).

Synthesis of M-WD (α_2_-K_X_P_2_MW_17_O_61_—Step 3): The lacunary POM doped with hetero addenda metals (M = Ru, Pd, and Pt) were synthesized by modification of previously published protocols: [33,34,35,36,37]. Initially, α_2_-K_10_P_2_W_17_0_61_ (α_2_-WD) (2.00 g, 0.42 mmol) was dissolved in 8 mL water at 90 °C. A solution of metal precursor (0.45 mmol, 1.1 equivalents) dissolved in 5 mL water at room temperature was then added dropwise to the α_2_-WD solution under vigorous stirring, after which the resulting solution was stirred at 90 °C for 1 h. KCl (1.19 g, 0.02 mmol) was subsequently dissolved in the reaction solution, which was allowed to cool to room temperature under continuous stirring for 1 h. The precipitate was collected by filtration and washed with cold millipore water followed by recrystallization in boiling water to give the α_2_-K_x_P_2_MW_17_O_61_ (M-WD). **Ru-WD**: Yield 3.19 g; ^31^P-NMR (162 MHz, D_2_O) δ/ppm: 5.06 (<0.01; impurity), −7.56 (0.09), −8.41 (0.12), −12.78 (2.00; Ru-WD), −13.89 (0.15), −14.01 (0.07), −14.07 (0.21), −14.33 (0.05), −15.66 (0.05). **Pd-WD**: Yield 3.19 g; ^31^P-NMR (162 MHz, D_2_O) δ/ppm: 5.06 (0.05; impurity), −6.99 (0.67; Pd-WD), −8.52 (0.21), −13.60 (0.01), −13.71 (0.02), −14.00 (0.23), −14.11 (2.00; Pd-WD). **Pt-WD**: Yield 3.22 g; ^31^P-NMR (162 MHz, D_2_O) δ/ppm: 5.06 (0.05; impurity), −6.98 (0.75; Pt-WD), −7.72 (0.01), −12.10 (<0.01), −12.36 (<0.01), −12.78 (0.05), −13.23 (<0.01), −13.72 (0.05), −13.82 (<0.01), −13.92 (<0.01), −14.00 (<0.01), −14.12 (2.00; Pt-WD).

### 2.3. Characterization of WD-POMs

The synthesized WD-POM materials were characterized by thermogravimetric analysis (TGA) on a Mettler Toledo TGA/DSC 1 with an aluminum sample holder. The measurements were carried out with nitrogen as carrier gas, with a heating rate of 5 °C/min up to 240 °C and hold for 1 h. ^31^P-NMR spectra were recorded at 105 MHz using a Bruker BioSpin GmbH spectrometer with a reference of H_3_PO_4_. Attenuated total reflectance (ATR) FTIR spectra were recorded on a Bruker ALPHA-P ATR-IR spectrophotometer (Billerica, MA, USA). Powder X-ray diffraction (XRD) was recorded on a Huber G670 Guinier (Rimsting, Germany) diffractometer using a Cu-Kα radiation from a focusing quartz monochromatic beam, with an exposure time of 1 h at an incident wavelength of 1.54 Å, in the 2θ interval of 3–100°. Single-crystal X-ray was recorded on a SuperNova Dual Atlas using CrysAlis PRO, Agilent Technologies (Santa Clara, CA, USA). X-ray fluorescence (XRF) was recorded on a PANalitical Epsilon3-XL instrument (Malvern Panalytical GmbH, Kassel, Germany) equipped with a high-resolution silicon drift detector and X-ray tube excitation (Rh, Ag, Mo anode materials, 20 kV, 100 μA). The element concentration was determined using the Omniant calibration program of the instrument, based on calibration lines of standards present in the instrument. The powder samples were analyzed directly in the apparatus. Inductive-coupled plasma (ICP-OES) analysis was carried out on a Perkin Elmer Plasma 400 (Waltham, MA, USA), using double distilled water as solvent.

### 2.4. Catalytic Hydrogenolysis of Glycerol with WD-POMs

Hydrogenation reactions of GL with M-WD POM catalysts were performed in a customized 10-fold screening plant in 20 mL stainless-steel high-pressure vessels equipped with magnetic stirring. For a typical reaction, water (10.0 mL), M-WD catalyst (0.10 g, 0.02 mmol active metal), and GL (1.00 g) were charged into the reaction vessel and the system was purged with hydrogen gas (three times) before the initial hydrogen pressure was adjusted to 50 bar. The stirring was then set to 300 rpm and the heating switched on. When the reaction reached the desired reaction temperature (150–250 °C), the stirring was adjusted to 1000 rpm. After the desired reaction time, the reactor was cooled down to room temperature and the liquid- and gas-phase subjected to analysis.

Catalyst recycling experiments were performed using GL (1.00 g), Ru-WD catalyst (0.10 g, 0.02 mmol active metal), and water (10 mL) in a 20 mL stainless-steel autoclave. The conditions were set to 50 bar H_2_, 200 °C, 1000 rpm, and were performed 24 h for each reaction run. After each run, new GL substrate (1.00 g) was added to the reaction solution.

### 2.5. Product Analysis

Gas-phase samples were collected from the reactors in gas sampling bags and analyzed with a Varian GC 450-TCD-FID equipped with a Shin Carbon ST column (2 m × 0.75 mm ID). Analysis of aliquots of liquid phases were carried out by means of ^1^H-NMR using dimethyl sulfoxide (DMSO) as an external standard in D_2_O allowed for identification of reaction products. GL conversion and product selectivities were calculated based on C-atoms (see Supporting Information, Equations (S1) and (S2)). The mass balance of the reactions was determined from ^1^H-NMR and GC data.

## 3. Results and Discussion

### 3.1. Catalytic Hydrogenolysis of Glycerol

#### 3.1.1. Catalyst Screening

**Scheme 1:**
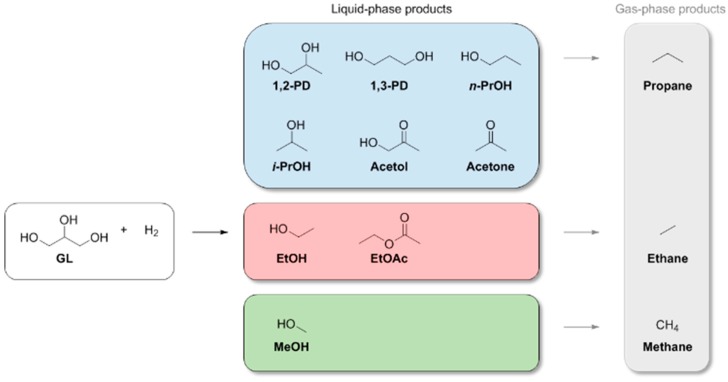
Observed products from the hydrogenolysis of glycerol.

Hydrogenolysis of glycerol in aqueous media was performed as an industrially relevant test reaction for comparison of the different noble metal-doped WD-POMs. Generally, the main products observed were C3 alcohols such as 1,2-PD, 1,3-PD, 1-propanol (*n*-PrOH), 2-propanol (*i*-PrOH) and ethanol (EtOH), together with the corresponding gaseous alkanes. Scheme 1 gives an overview of the typical products observed during GL hydrogenolysis [6,12].

The results of the initial catalyst screening are compiled in Table 1, comparing the synthesized WD-POMs with their water-soluble metal precursors (RuCl_3_, PdCl_2_, and H_2_PtCl_6_) and the commercial heterogeneous supported catalysts, Ru/C, Pd/C, and Pt/C, respectively. Notably, equal amounts of noble metal (0.2 mmol) were used in each series in order to allow direct comparison of the different catalyst activities.

The unsubstituted WD-POMs (entries 10 and 11) as well as a reference experiment without any catalyst (entry 12) resulted in essentially no GL conversion. Moreover, the Pt- and Pd-containing WD catalysts, as well as the metal precursors and the carbon-supported heterogeneous analogues, resulted in very low GL conversions (1%–7%, entries 4–9). While the Pd-WD and the Pd/C afforded the highest selectivities for 1,2-PD, PdCl_2_ gave predominantly *n*-PrOH as the liquid-phase product. For the different Pt species, the main product was also 1,2-PD for the Pt-WD and Pt/C, while H_2_PtCl_6_ gave predominantly *n*-PrOH.

The highest activities towards GL conversion were obtained applying the Ru-based catalysts, however also here, different selectivities were observed for the different Ru species. These results are in good agreement with similar systems reported by Dasari et al. [13]. The Ru-WD catalyst afforded the highest GL conversion (26%) leading mainly to the formation of 1,2-PD in the liquid phase (entry 1). In comparison, RuCl_3_ and Ru/C (entries 2 and 3) both provided lower GL conversion (17%) and favored formation of the undesired methane byproduct (44%). This strongly indicates a direct relationship between catalytic structure and product selectivity. This is interestingly accompanied by only a mild acidity (pH = 5.6) of the reaction solution containing the Ru-WD catalyst. As expected, the RuCl_3_ precursor showed the highest acidity of all used Ru catalysts resulting in a pH value of 3.1. Moreover, also Ru/C showed a higher acidity (pH = 4.1) than the Ru-WD catalyst, which obviously negatively affected both activity and selectivity.

**Scheme 2:**
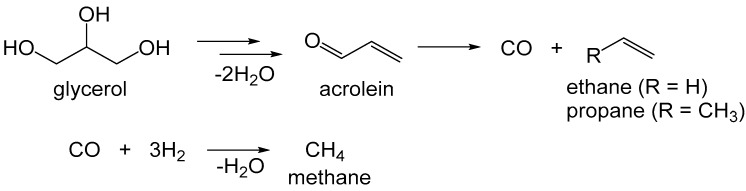
Undesired reaction pathway using non-acidic Ru catalysts [6,15].

Concerning the different product selectivities obtained for the different Ru catalysts, both the water-soluble homogeneous RuCl_3_ and the heterogeneous Ru/C mainly catalyzed the undesired methanation reaction resulting from non-specific hydrogenolysis of glycerol to methane, possibly following the reaction pathway suggested by ten Dam and Hanefeld (Scheme 2) [6]. Here, initial thermal-induced dehydration to acrolein takes place followed by decomposition to carbon monoxide and short-chain alkanes (ethane, propane), which was detected in small amounts during the experiments. Subsequently, the formed CO reacts at the active sites of the Ru catalysts leading to methanation. 

**Scheme 3:**
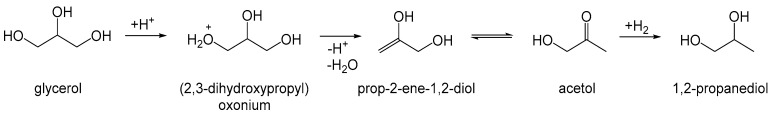
Desired reaction pathway using the acidic Ru-WD catalyst [14].

In comparison, the Ru-WD catalyst predominantly formed the desired 1,2-PD as a main reaction product likely resulting from the suggested reaction sequence by Huang et al. (Scheme 3) [14].

The Ru-WD POM provides several acidic sites that can initialize the first protonation step followed by acid- and thermally induced consecutive dehydration and subsequent keto-enol tautomerization leading to acetol as reaction intermediate. In the following step, selective hydrogenation to 1,2-PD takes place [6]. This reaction sequence is generally accepted as kinetically controlled as acetol is a relatively stable intermediate [6,7,14].

#### 3.1.2. Catalysis with Ru-WD

##### Effect of Glycerol Concentration, Temperature, and Reaction Time

**Figure 2:**
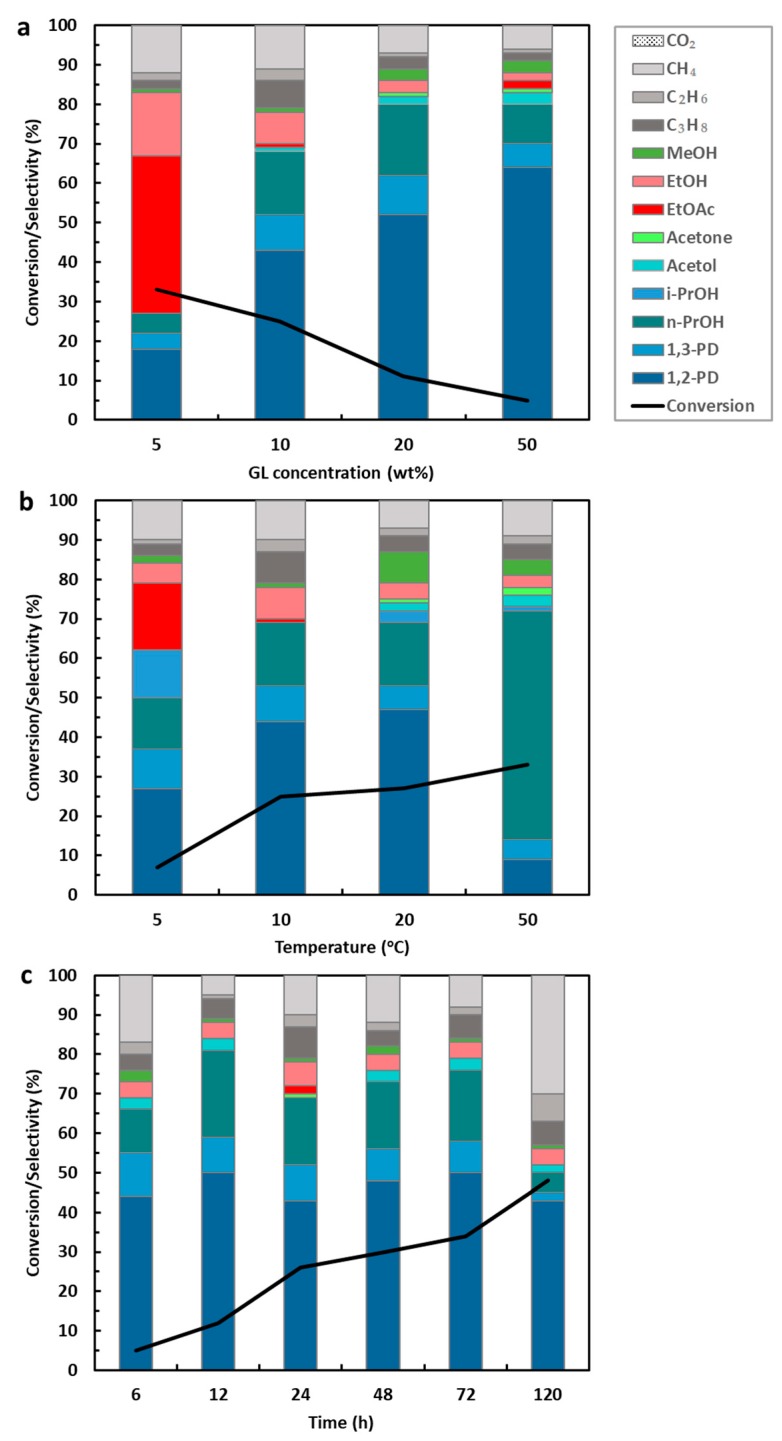
GL conversions and product selectivities with different (**a**) GL concentrations, (**b**) temperatures, and (**c**) reaction times. General reaction conditions: 0.5–5.0 g GL (6–55 mmol) (corresponding to 5–50 wt.%), 0.1 g Ru-WD (0.02 mmol Ru), 10 mL H_2_O, 50 bar H_2_, 150–250 °C, 6–120 h, 1000 rpm.

Based on the initial screening results, the best-performing catalyst Ru-WD was selected for further studies, where the influence of various reaction parameters on the catalyst performance was investigated. First, the effect of GL concentration in water was examined (see Figure 2a). 

Higher dilution led to higher total GL conversion and the highest conversion of 33% was achieved using 5 wt.% GL in water. However, these data indicate that GL had a negative reaction order as higher GL concentrations led to lower reaction rates, which can be deduced from Figure 2a as all experiments were run for the same reaction time. Moreover, the selectivity shifted from favoring 1,2-PD at GL concentrations above 10 wt.% to yield ethyl acetate (EtOAc) as the main product at the lowest concentration of 5 wt.%. Interestingly, only two liquid byproducts (*n*-PrOH and 1,3-PD) with selectivities above 10% were observed, while only traces of all other byproducts were obtained. Regarding the gas phase, methane selectivity increased with increasing conversion. These observations led to the assumption that different reaction pathways are preferred at different GL concentrations as previously reported by Dasari et al. for similar systems [13].

The effect of different reaction temperatures at an optimum GL loading of 10 wt.% was further investigated. Figure 2b shows that the conversion of GL increased with increasing temperatures (as expected) from 7% at 150 °C up to 33% at 250 °C. However, the selectivity also changed depending on the reaction temperature. At 150 °C, the major liquid products were 1,2-PD (28%), EtOAc (17%), *n*-PrOH (13%), and *i*-PrOH (12%), and the main gaseous product was methane (11%). All other products were observed in selectivities below 10%. With increasing temperature, and thus conversion, the selectivity for 1,2-PD increased up to 46% at 225 °C. The only other product formed above 150 °C was *n*-PrOH (18% selectivity). This trend did not continue at higher temperatures and at 250 °C, the selectivity shifted drastically towards *n*-PrOH as the main product (59%), while the selectivity for 1,2-PD decreased to 10%. Hence, 225 °C was found to be the optimum temperature for the reaction system, giving the best compromise between GL conversion and 1,2-PD yield.

Further, the kinetics of the reaction system was investigated under the aforementioned optimized conditions (see Figure 2c). Six individual experiments were performed with reaction times between 6–120 h at 225 °C with 10 wt.% GL solution. The results indicated a general trend of increasing conversion with increasing reaction time, affording a GL conversion of 50% after 120 h. The long reaction times suggested the catalytic system to be slow under the applied reaction conditions, probably associated with a high activation energy of the Ru-WD catalyst. Importantly, a continuous increase in 1,2-PD selectivity up to 51% was observed after 72 h, together with decreasing amounts of byproducts except for *n*-PrOH, where a maximum selectivity of 16% was obtained after 72 h. However, after 120 h, the selectivity of methane increased drastically reaching a maximum of 28%, while the 1,2-PD selectivity slightly decreased to 43%, implying that the complete hydrogenolysis of GL to methane was favored at longer reaction times.

Notably, 1,2-PD was the main product with all variations in reaction parameters giving selectivities up to 50% with *n*-PrOH as the only major byproduct in the liquid phase. The two products should be easily separated by distillation, as the boiling point of *n*-PrOH (97 °C) is far below that of 1,2-PD (188 °C). Consequently, downstream processing is potentially easy in terms of upscaling the process to a technical scale.

##### Effect of Catalyst Concentration, Hydrogen Pressure, and Stirring Speed

**Figure 3:**
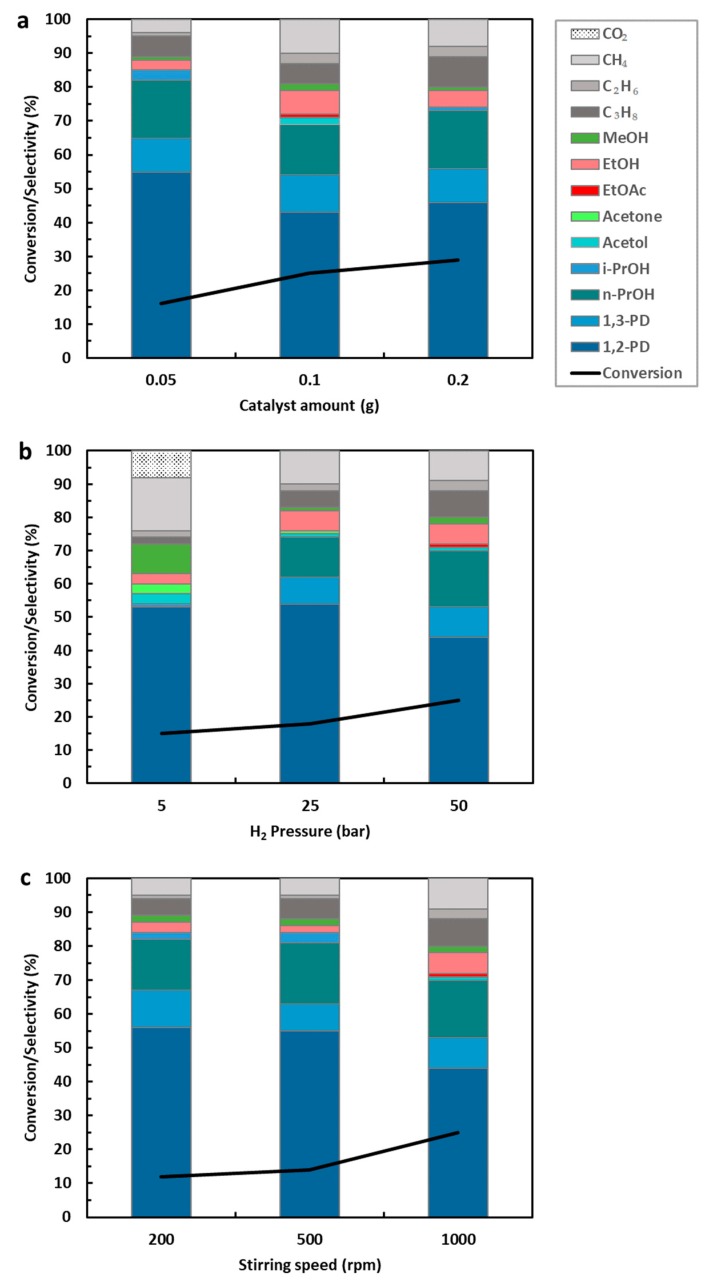
GL conversions and product selectivities with different (**a**) catalyst amount, (**b**) H_2_ pressure, and (**c**) stirring speed. General reaction conditions: 1.0 g GL (11 mmol) (corresponding to 10 wt.%), 0.05–0.2 g Ru-WD (0.01–0.04 mmol Ru), 10 mL H_2_O, 5–50 bar H_2_, 200 °C, 24 h, 200–1000 rpm.

In the next set of experiments, the catalyst amount was altered in order to validate if the GL conversion could be increased by higher catalyst loading. The results in Figure 3a show that higher catalyst concentrations indeed led to higher GL conversion. However, the effect was not directly proportional to the added catalyst amount as only a 100% increase (15% to 30% GL conversion) was monitored with four times catalyst amount added, suggesting that not all the Ru inventory was catalytically active. Another interesting observation was that, also here, increasing GL conversion seemed to promote the undesired non-acidic pathway (Scheme 2) resulting in higher methane and ethane selectivities compared to the desired acidic reaction pathway (Scheme 3) leading to the desired propanediols.

In order to assess if the catalytic reactions were performed in a kinetic or mass transport controlled regime, both hydrogen pressure (Figure 3b) and stirring speed (Figure 3c) were varied to evaluate if hydrogen availability in the liquid reaction phase was rate limiting for the overall reaction. As expected, the GL conversion increased with increasing hydrogen pressure under otherwise constant reaction conditions, due to the low solubility of hydrogen in aqueous solutions especially at the higher temperatures applied (i.e., mass transport controlled). Regarding product selectivity, a slightly higher 1,2 PD selectivity was obtained at lower hydrogen pressures of 5–25 bar compared to 50 bar. Moreover, with the lowest hydrogen pressure methanol, acetone and CO_2_ formed as main side products beside methane, possibly by competing retro-aldol condensations and water-gas shift reaction, respectively. In contrast, *n*-PrOH and 1,3-PD were prevailing side products at higher H_2_ pressure, indicating that the desired acidic reaction pathway (Scheme 3) was favored under higher hydrogen pressures.

When hydrogenolysis was conducted at varied stirring speed, it allowed evaluation of the potential influence of film diffusion during reaction. The results plotted in Figure 3c show that increased GL conversion, and thus increased reaction rate (as a constant reaction time was applied), were obtained when the reaction was completed with 1000 rpm (highest possible stirring speed for the applied reaction setup) compared to lower stirring speeds. This strongly indicates that lower stirring speeds (<500 rpm) led to a film diffusion limited regime. 

##### Catalyst Reuse

The reusability of the Ru-WD catalyst was examined using reaction conditions of 50 bar H_2_ and 200 °C. The results shown in Table 2 confirmed that the catalyst largely maintained its initial selectivity after three recycling runs, whereas lower total GL conversion for each reaction run (from 26% to 10%) suggested decreased catalyst activity. However, the initial GL concentration in each experiment increased gradually due to incomplete conversion with substrate successively added, and conversion alone did therefore not give a fair comparison. Thus, for the second reaction run where 1.0 g GL substrate was added to the remaining solution from the first run containing 74% unconverted GL (i.e., 0.74 g), the initial substrate concentration was increased from 10 to almost 18 wt.%. Similarly, the initial GL concentration in the third reaction was around 25 wt.% after addition of another 1.0 g of fresh GL to the solution after the second run. Taking the difference in GL concentrations into account and reporting the total amount of converted GL for each run revealed that the same quantity of GL was converted in each run, demonstrating that the catalyst activity remained constant. Hence, overall, the results found in the recycling experiments are well in line with the results obtained at the same GL concentration (Figure 2a), and 10% GL conversion in the third recycling run does not imply catalyst deactivation. 

#### 3.1.3. Characterization of WD-POMs

Ru-, Pd-, and Pt-doped α_2_-WD POMs (Table 3) were prepared by a three-step procedure from α-WD POM and structural characterized by ^31^P-NMR, ATR-FTIR, and powder XRD. Elemental analysis of the materials was further provided from XRF and ICP analysis and the content of hydration water calculated based on TGA results (Supporting Information, Figures S1–S11). 

##### Structural Analysis by ^31^P-NMR, ATR-FTIR, and XRD

The ^31^P-NMR spectra of the parent α- and α_2_-WD materials (Supporting Information, Figures S1 and S2) were in good accordance with the literature [30,31,32,33]. Also, the spectra of the metal-doped WDs compared well to literature data despite minor impurities that were observed in the spectra of the latter (Supporting Information, Figures S3–S5). Thus, for Ru-WD, only one major ^31^P peak was observed for P(2) (δ −12.1 ppm) in the structure, since the paramagnetic nature of Ru(III) shift and broaden the NMR signal for P(1) enabling only the phosphor atom positioned furthest away from Ru to be observed [34,35]. Moreover, the nuclear quadrupole moments of ^99^Ru and ^101^Ru allowed fast relaxation of P(1) resulting in only one sharp peak for P(2) [34]. In contrast, Pd(II) is diamagnetic and doping of Pd(II) into α_2_-WD was expected to afford two inequivalent peaks corresponding to a broadened peak for P(1) closest to the phosphor (caused by fast relaxation) and a sharp peak for P(2) at δ −11.9 ppm [34,36]. Instead, two sets of peaks were observed in the spectrum for the Pd-WD (δ 8.52 and −14.00 ppm) with the major peak being comparable to parent α_2_-WD and the minor peaks comparable to the Pd-WD dimeric species, [syn-Pd_2_(α_2_-P_2_W_17_O_61_H_3_)_2_]^10−^ and [anti-Pd_2_(α_2_-P_2_W_17_O_61_H_0.5_)_2_]^15−^ with chemical shifts separated by only 0.1 ppm. [37] Like Pd(II), Pt(II) is also diamagnetic and two inequivalent peaks were observed (δ −7.0 and −14:0 ppm) for the Pt-doped WD as expected. However, since the peaks overlapped the regions of the α_2_-WD, the NMR data alone were not sufficient to confirm Pt incorporation into the POM.

**Figure 4:**
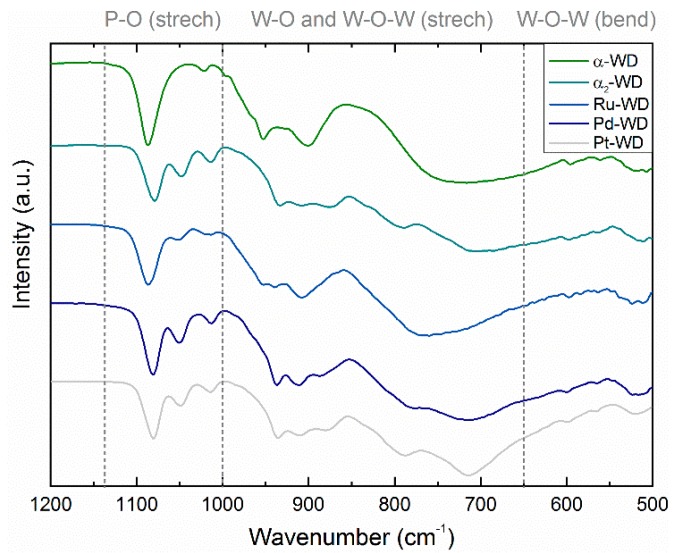
ATR-FTIR spectra of the prepared α-WD, α_2_-WD and metal-doped WDs in the range 1200 to 500 cm^−1^.

Additional structural information was obtained for the WDs materials from ATR-FTIR spectra of the compounds, where also the IR spectra of the parent α-WD and α_2_-WD (Figure 4) were in good agreement with the literature [38,39,40,41,42]. The α-WD was characterized by three stretching bands in the P-O region at 1087 cm^−1^ (strong), 1021 cm^−1^ (weak), and 996 cm^−1^ (shoulder) and W-O-W stretching bands at 953 cm^−1^ (terminal W-O), 901 cm^−1^ (“inter” bridge between corner-sharing octahedra), and 733 cm^−1^ (“intra” bridge between edge-sharing octahedra) as well as W-O-W bending vibrations in the 600–500 cm^−1^ (weak) region. Removal of a tungsten from α-WD to give α_2_-WD caused splitting of the band at 1087 cm^−1^ into three distinct stretching bands at 1079, 1047, and 1013 cm^−1^, respectively, due to the absence of bonding interactions between P(1) and the removed tungsten octahedra. Furthermore, the asymmetry of α_2_-WD caused small displacements of the W-O stretch (933 cm^−1^), the “inter” W-O-W (875 cm^−1^) and the “intra” W-O-W (714 cm^−1^) bands. For the prepared Ru- and Pd-doped WDs analogous bands in the characteristic α_2_-WD regions were observed with small displacements due to metal substitution into the lacuna in agreement with literature. However, the IR spectrum for the Pt-WD was nearly identical to the spectrum for the α_2_-WD, suggesting that Pt was not efficiently substituted into the lacuna but possibly coordinated as a counter cation (like potassium cations) or not associated with the WD structure [43,44]. 

**Figure 5:**
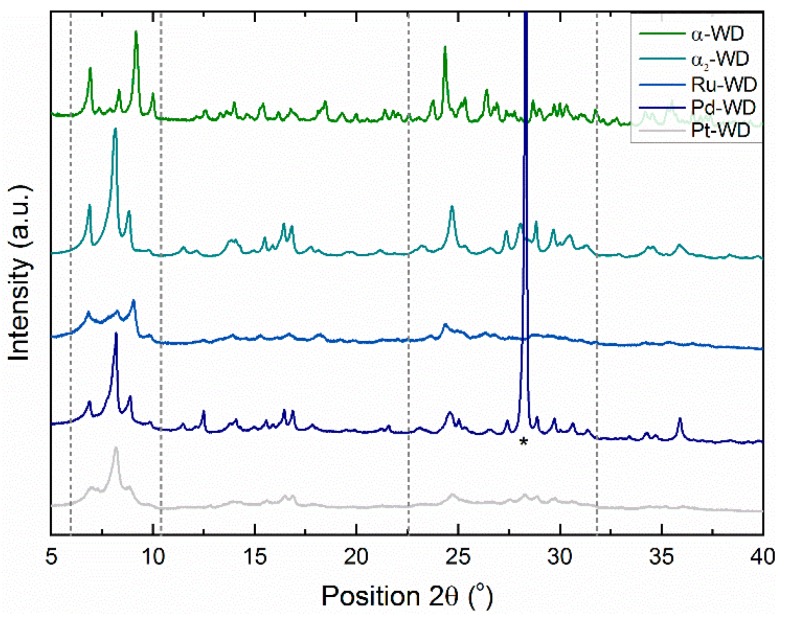
Powder XRD of the prepared WD materials (* indicates KCl impurity).

A single-crystal X-ray structure determination (Supporting Information, Figure S6 and Tables S1 and S2) of the prepared α-WD confirmed its structure [32,33], as also suggested by the information obtained from ^31^P-NMR and IR. Unfortunately, crystals with sufficient size and quality were not obtained for the α_2_- and metal-doped WD catalysts to allow similar structure determination, and these were analyzed by powder XRD as an alternative (Figure 5). The diffractogram of α_2_-WD was in good agreement with data found in literature [44,45], and characteristic peaks in the α_2_-region (6–10° and 22–32°) were clearly observed for the Pd- and Pt-WD materials indicating preservation of the α_2_-WD structure. In contrast, the peak pattern of Ru-WD was less clear, making comparison of the specific peak patterns challenging. 

##### Elemental Analysis by XRF and ICP

Elemental compositions of the prepared WD compounds were measured by XRF and ICP-OES and the results are compiled in Table 4. In general, slightly higher W/P and K/P ratios were found by XRF analysis compared to the theoretical ratios for the metal-doped WDs, whereas comparably lower ratios were measured by ICP possibly due to partial precipitation of the WD compounds during the latter analysis. Notably, the metal-doped WDs all contained close to the expected M/P ratios indicating successful cation substitution (but not necessarily metal incorporation into the structure). Minor impurities of Cl (likely as chloride) were found in α-, α_2_-, Ru-, and Pd-WDs by the XRF analysis (results not shown), and an excess of K was further observed in the ICP analysis of Pt-WD indicative of KCl impurity, as also found by the XRD (Figure 5).

## 4. Conclusions

Well–Dawson POMs doped with the noble metals Ru, Pt, and Pd were prepared, characterized by ^31^P-NMR, FTIR, XRD, and XRF/ICP, and applied as novel homogeneous catalysts for the selective hydrogenolysis of aqueous glycerol to propanediols. The Ru-WD catalyst showed superior catalytic performance towards formation of the desired product 1,2-PD with the reaction temperature, glycerol concentration, hydrogen pressure, as well as stirring speed playing important roles for both the product selectivity and the conversion, indicating that the reaction was mass transfer controlled. Importantly, the Ru-WD POM proved reusable in three consecutive reactions performed under optimized conditions without exhibiting any apparent deactivation. This potentially makes the catalyst system prone for technical applications, however further improvement in reaction rates and product yields are necessary in order to compete with state-of-the-art industrial catalysts.

## Figures and Tables

**Table 1:** Catalytic performance of WD-based catalysts and other metal-based catalysts in glycerol (GL) hydrogenolysis ^a^.

Entry	Catalyst	Conversion (%) ^b^	pH ^c^	Product Selectivity in Phases (%)							
				Liquid ^b^						Gas ^d^
				1,2-PD	1,3-PD	*n*-PrOH	EtOAc	Acetone	Acetol	CH_4_
1	Ru-WD	26	5.6	42	9	17	1	0	1	10
2	RuCl_3_	17	3.1	17	4	12	2	0	0	44
3	Ru/C ^e^	17	4.1	31	1	2	0	0	0	44
4	Pd-WD	1	5.9	60	0	14	0	0	7	1
5	PdCl_2_	3	2.7	16	19	59	0	0	0	0
6	Pd/C ^e^	2	3.4	74	0	6	0	0	0	0
7	Pt-WD	3	6.0	63	15	8	0	2	1	0
8	H_2_PtCl_6_	7	2.5	13	18	57	0	0	0	6
9	Pt/C ^e^	6	4.1	78	6	6	0	0	0	0
10	α-WD	1	6.4	8	0	5	39	8	14	1
11	α_2_-WD	0	6.3	18	0	0	0	18	35	1
12	none	0	7.0	0	0	0	0	0	0	0

^a^ Reaction conditions: Glycerol (1.0 g), catalyst (0.02 mmol active metal), water (10 mL), 50 bar H_2_, 200 °C, 24 h, 1000 rpm; small amounts of i-PrOH, EtOH, MeOH, propane, and ethane were also observed in most of the experiments. ^b^ GL conversion and liquid-phase selectivities were determined by ^1^H-NMR. ^c^ pH value of solution after the reaction. ^d^ Gas-phase selectivities were determined by GC. ^e^ 5 wt.% metal on activated carbon.

**Table 2:** Recycling of Ru-WD catalyst in GL hydrogenolysis ^a^.

Entry	Conversion (%) ^b^	Converted GL (g)	Product Selectivity in Phases (%)											
			Liquid ^b^									Gas ^c^		
			1,2-PD	1,3-PD	*n*-PrOH	EtOAc	Acetone	Acetol	EtOH	MeOH	CH_4_		C_2_H_6_	C_3_H_8_
1	26	0.26	42	9	17	1	0	1	7	1	10		3	7
2 ^d^	14	0.24	49	11	20	2	0	0	6	1	4		1	4
3 ^e^	10	0.25	54	7	14	0	0	0	8	3	6		2	5

^a^ Reaction conditions: Glycerol (1.0 g), catalyst (0.02 mmol active metal), water (10 mL), 50 bar H_2_, 200 °C, 24 h, 1000 rpm; small amounts of i-PrOH, EtOH, MeOH, propane and ethane were also observed in most of the experiments. ^b^ GL conversion and liquid-phase selectivities were determined by ^1^H-NMR; ^c^ Gas-phase selectivities were determined by GC. ^d^ Second reaction run. ^e^ Third reaction run.

**Table 3:** Overview of prepared WD-POMs.

Material	Abbreviation	Color	Water Content (mol%) ^a^
α-K_6_P_2_W_18_O_62_	α-WD	Yellow	8
α_2_-K_10_P_2_W_17_O_61_	α_2_-WD	White	16
α_2_-K_7_P_2_W_17_O_61_Ru	Ru-WD	Black	9
α_2_-K_8_P_2_W_17_O_61_Pd	Pd-WD	Brown	8
α_2_-K_8_P_2_W_17_O_61_Pt	Pt-WD	Light brown	11

^a^ Determined by TGA after recrystallization and drying.

**Table 4:** XRF and inductive-coupled plasma (ICP-OES) results of the synthesized WD materials ^a^.

Material	W/P Ratio (mol/mol)			K/P Ratio (mol/mol)			M/P Ratio (mol/mol) ^b^	
	XRF	ICP	Theo.	XRF	ICP	Theo.	ICP	Theo.
α-WD	9.1	8.8	9.0	3.0	2.3	3.0	0.0	0.0
α_2_-WD	8.4	8.1	8.5	5.1	4.4	5.0	0.0	0.0
Ru-WD ^c^	8.6	8.1	8.5	4.1	5.2	3.5	0.7	0.5
Pd-WD ^d^	9.2	8.1	8.5	5.2	3.9	4.0	0.4	0.5
Pt-WD ^d^	9.9	8.3	8.5	5.2	8.0	4.0	0.3	0.5

^a^ The raw XRF data were converted into molar concentrations relative to phosphorus based on a calibration curve calculated from α- and α_2_-WD (see Supporting Information, Figures S7–S11). ^b^ M = doped metal. ^c^ Recrystallized twice from boiling water. ^d^ Recrystallized from methanol.

## Supplementary Materials

The following are available online at https://www.mdpi.com/1996-1944/12/13/2175/s1, Figure S1: ^31^P NMR spectrum of **α-WD**, **Figure S2**: ^31^P NMR spectrum of **α_2_-WD, Figure S3**: ^31^P NMR spectrum of **Ru-WD, Figure S4**: ^31^P NMR spectrum of **Pd-WD, Figure S5**: ^31^P NMR spectrum of **Pt-WD, Figure S6**: Single crystal X-ray diffraction of α-WD. (a) Side view of one α-WD, (b) top view of unit cell, (c) side view of unit cell and (d) polyhedral representation of crystal structure. Oxygen (red), potassium (green), WO_6_ octahedra (pink) and PO_4_ tetrahedra (blue), Figure S7: XRF spectrum of **α-WD, Figure S8**: XRF spectrum of **α_2_-WD, Figure S9**: XRF spectrum of **Ru-WD, Figure S10**: XRF spectrum of **Pd-WD,** Figure S11: XRF spectrum of **Pt-WD, Figure S12**: Calibration curve based on data presented in Table S3, Table S1: Overview of single crystal XRD data. ^a^ Dawson 1953^96^, ^b^ ICSD—Inorganic Crystal Structure Database^121^, Table S2: Single Crystal XRD data. Computer programs: CrysAlis PRO, Agilent Technologies, Version 1.171.37.34 (release 22-05-2014 CrysAlis171 .NET) (compiled May 22 2014, 16:03:01), SHELXS (Sheldrick, 2008), SHELXL (Sheldrick, 2015), Olex2 (Dolomanov et al., 2009), Table S3: Raw XRD data acquired (counts) and calculated ratios thereof.
