# Supplementary material for: Ru-Doped Wells–Dawson Polyoxometalate as Efficient Catalyst for Glycerol Hydrogenolysis to Propanediols
Source: Materials (Basel). 2019 Jul 6;12(13):2175. doi: 10.3390/ma12132175 (PMC6651741; doi:10.3390/ma12132175)
Supplement: Supplementary file 1 [file materials-12-02175-s001.pdf]

materials

Article

Ru-Doped Wells-Dawson Polyoxometalate as Efficient Catalyst for Glycerol Hydrogenolysis to Propanediols

Amalie Modvig [1], Chiraphat Kumpidet [2], Anders Riisager [1] and Jakob Albert [2,*]

[1] Centre for Catalysis and Sustainable Chemistry, Department of Chemistry, Technical University of Denmark, DK-2800 Kgs. Lyngby, Denmark; ammod@kemi.dtu.dk (A.M.); ar@kemi.dtu.dk (A.R.)
[2] Lehrstuhl für Chemische Reaktionstechnik, Friedrich-Alexander-Universität Erlangen-Nürnberg, Egerlandstr. 3, 91058 Erlangen, Germany; Chiraphat.kumpidet@fau.de
* Correspondence: jakob.albert@fau.de

Supporting Information

$^{31.}$P NMR Spectra

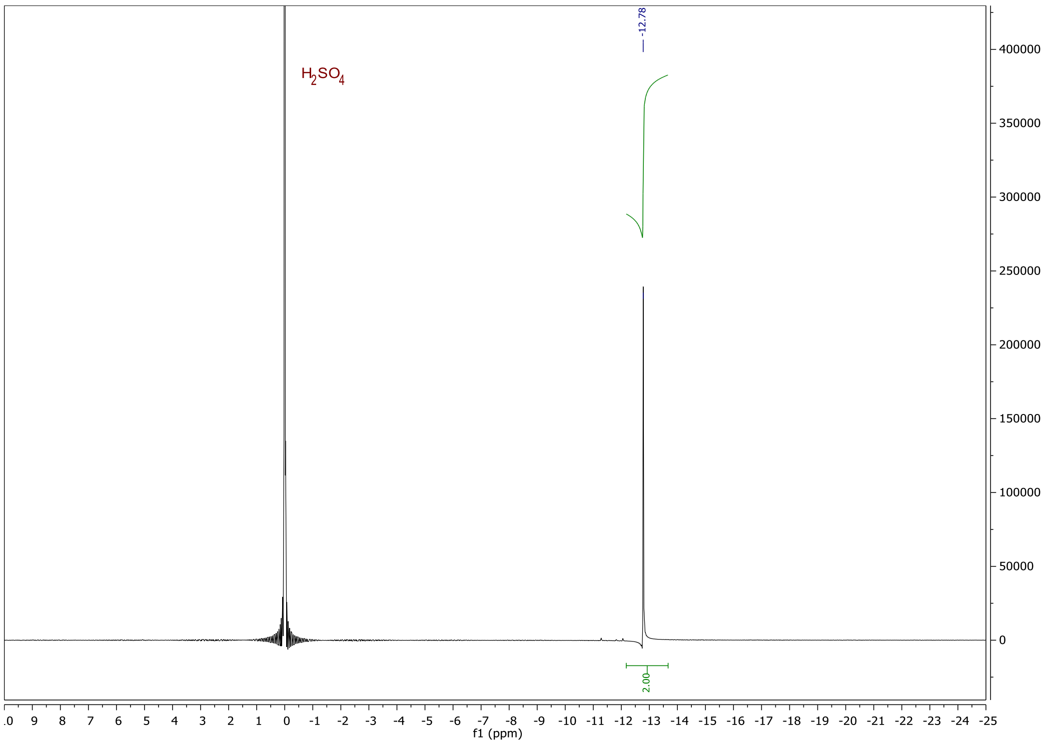

Figure S1. ^{31}P NMR spectrum of ***α*-WD.**

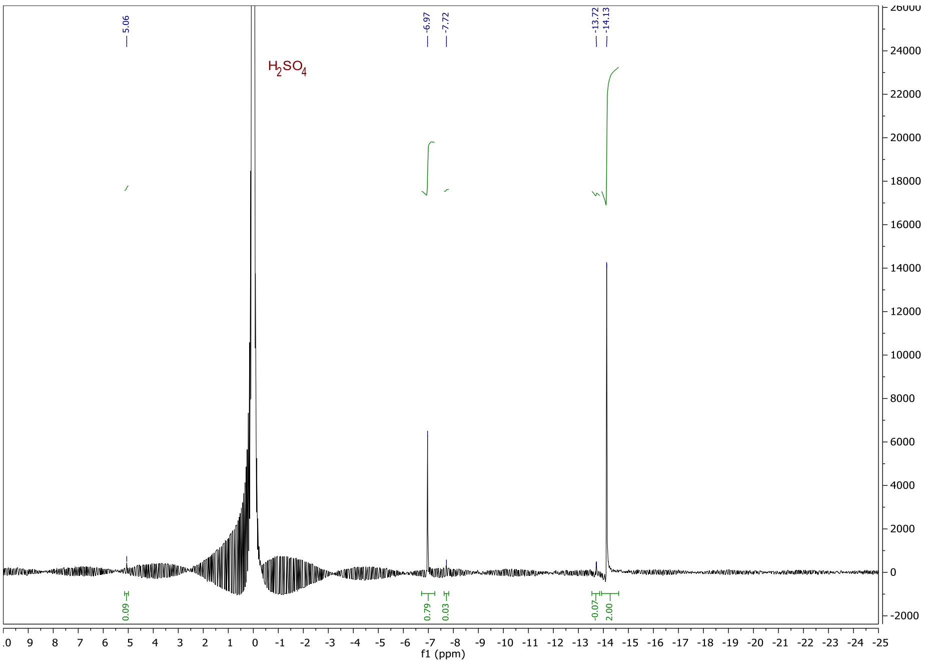

Figure S2. ^{31}P NMR spectrum of α_2**-WD.**

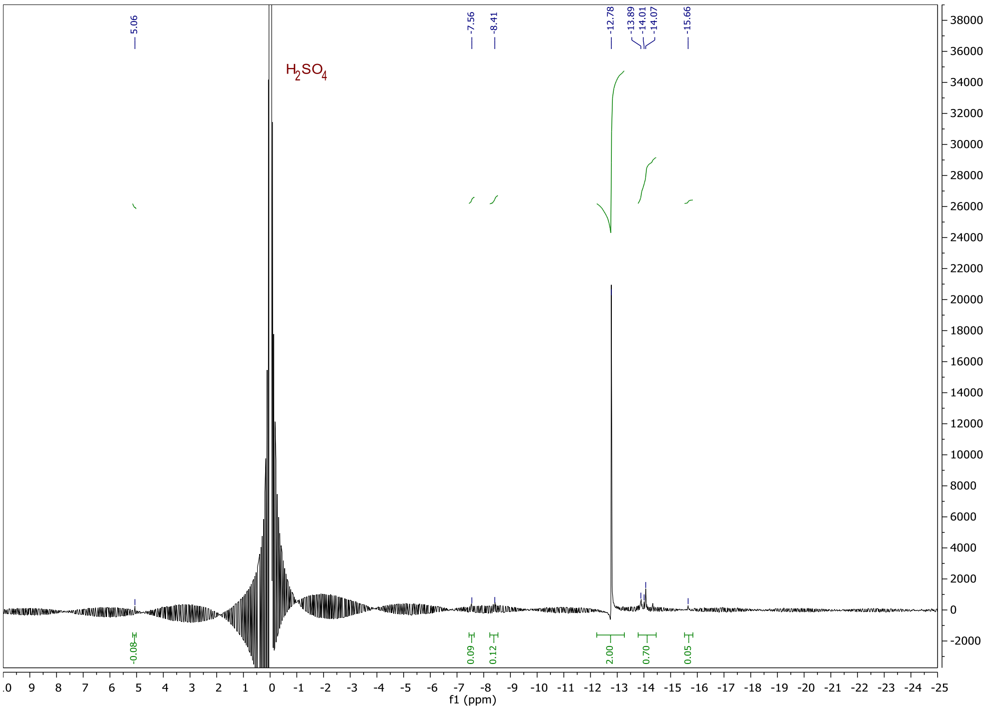

Figure S3. ^{31}P NMR spectrum of **Ru-WD.**

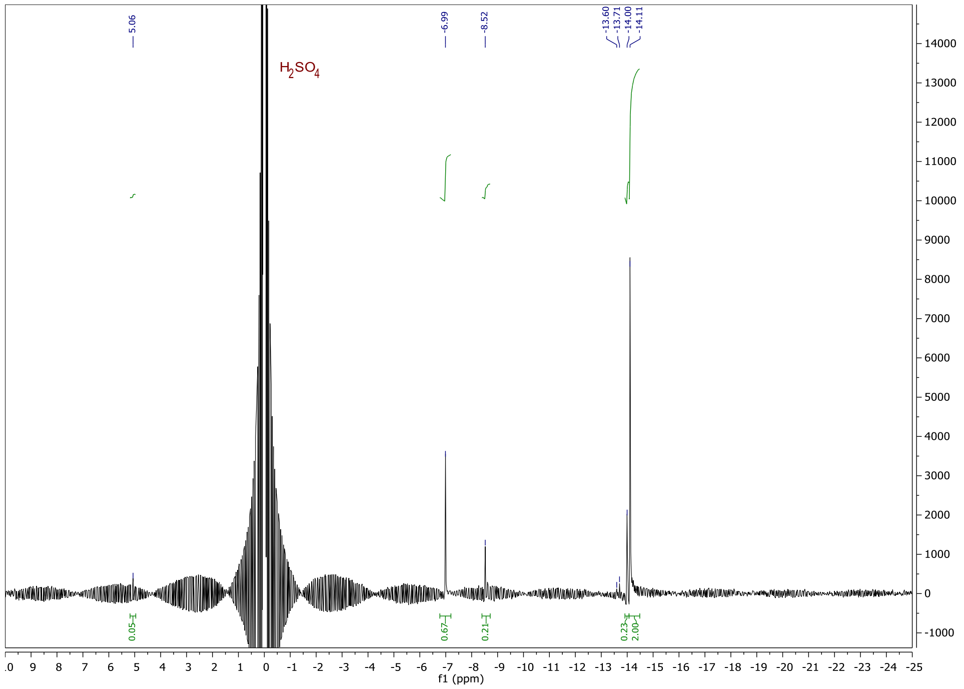

Figure S4. ^{31}P NMR spectrum of **Pd-WD.**

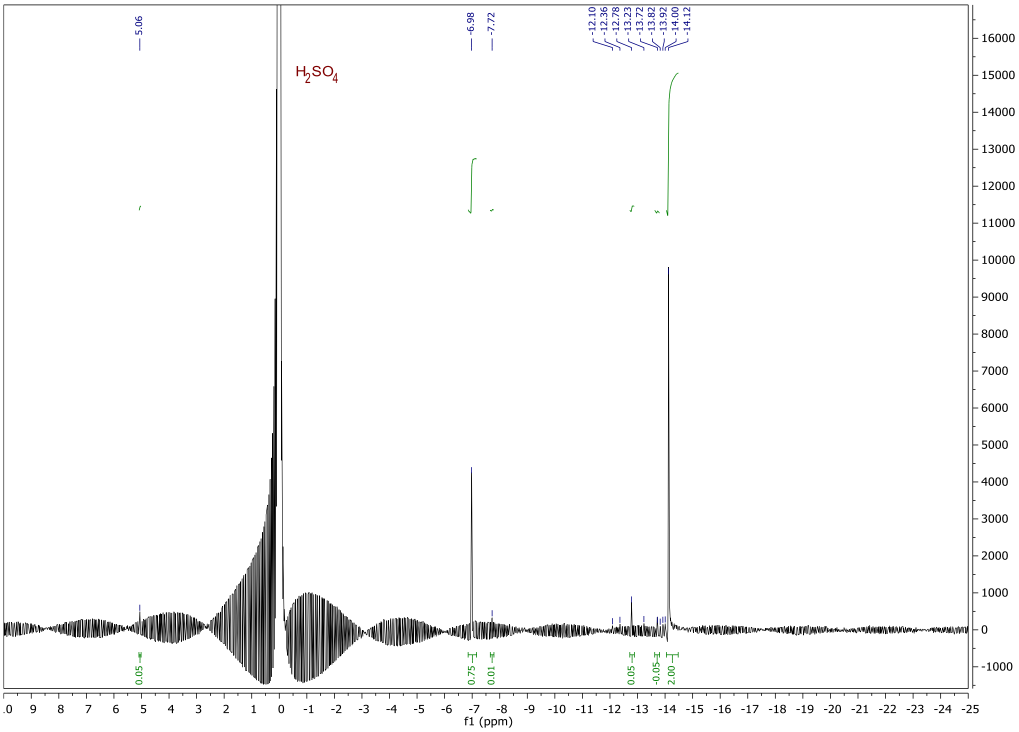

Figure S5. ^{31}P NMR spectrum of **Pt-WD.**

Single Crystal XRD

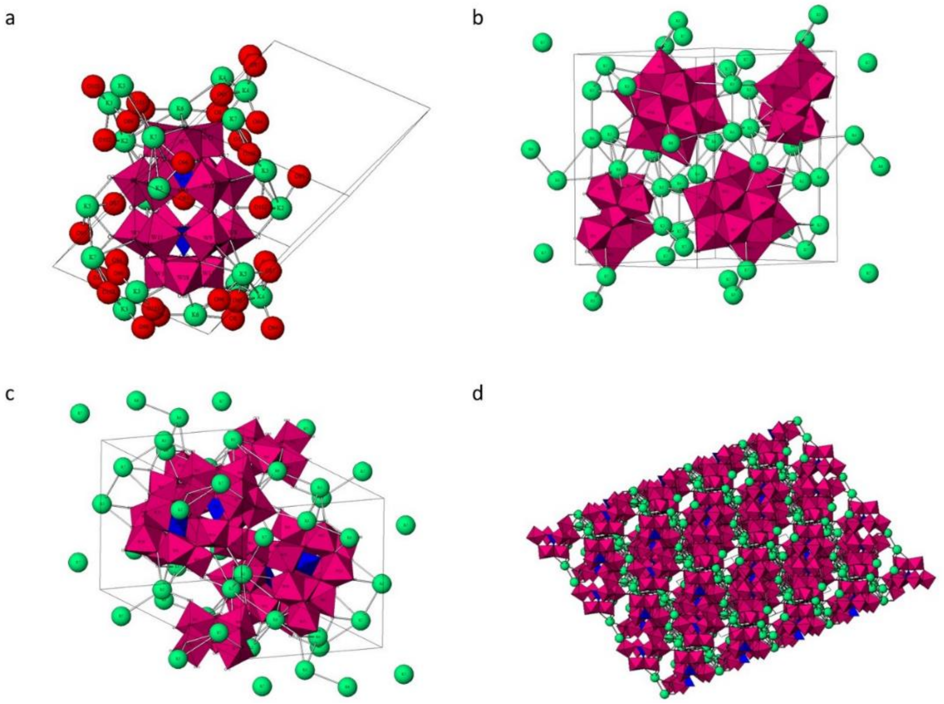

Figure S6. Single crystal X-ray diffraction of α-WD. (**a**) Side view of one α-WD, (**b**) top view of unit cell, (**c**) side view of unit cell and (**d**) polyhedral representation of crystal structure. Oxygen (red), potassium (green), WO_6 octahedra (pink) and PO_4 tetrahedra (blue).

Table S1. Overview of single crystal XRD data. [a] Dawson 1953[96], [b] ICSD—Inorganic Crystal Structure Database[121].

Crystal Data	α-WD (synthesized)	α-WD (Dawson) [a]	Standardized Structure [b]
Chemical formula	$K_6O_{72}P_2W_{18}$	$K_6O_{72}P_2W_{18}$	$K_6O_{72}P_2W_{18}$
Space group	Triclinic, *P*-1	Triclinic, *P*-1	Triclinic, *P*-1
a (Å)	12.79973	12.8612	12.8600
b (Å)	14.80863	14.8315	14.8300
c (Å)	18.62205	22.3422	20.1187
α (°)	101.2082	94.4033	68.8150
β (°)	96.8322	116.8(33	82.1060
γ (°)	115.1402	115.6033	64.4000
V (Å³)	3052.62	3225.59	3225.60

Table S2. Single Crystal XRD data. Computer programs: CrysAlis PRO, Agilent Technologies, Version 1.171.37.34 (release 22-05-2014 CrysAlis171 .NET) (compiled May 22 2014, 16:03:01), SHELXS (Sheldrick, 2008), SHELXL (Sheldrick, 2015), Olex2 (Dolomanov et al., 2009).

**Crystal Data**	**α-WD**
Chemical formula	$K_6O_{72}P_2W_{18}$
M_r	4757.84
Crystal system, space group	Triclinic, *P*1
Temperature (K)	299
a, *b*, *c* (Å)	12.7997 (3), 14.8086 (3), 18.6220 (5)
α, β, γ (°)	101.208 (2), 96.832 (2), 115.140 (2)
V (Å^3)	3052.62 (15)
Z	2
Radiation type	Mo *K*α
D_c (gcm^{-3})	5.176
μ (mm^{-1})	34.35
F(000)	4104
2θ range (°)	3.16 – 29.39
Data collection	
T_{min}, T_{max}	0.287, 1.000
No. of measured, independent and observed [$I > 2\sigma(I)$] reflections	50784, 15022, 12790
R_{int}	0.060
$(\sin \theta/\lambda)_{max}$ (Å^{-1})	0.695
Refinement	
$R[F^2 > 2\sigma(F^2)]$, $wR(F^2)$, S	0.043, 0.102, 1.12
No. of reflections	15022
No. of parameters	883
$\Delta\rho_{max}$, $\Delta\rho_{min}$ (e Å^{-3})	2.70, −3.67
Limiting indices	$-17 \leq h \leq 15$, $-19 \leq k \leq 20$, $-25 \leq l \leq 25$
Diffractometer	SuperNova, Dual, Cu at zero, Atlas
Absorption correction	Multi-scan *CrysAlis PRO*, Agilent Technologies, Version 1.171.37.34 (release 22-05-2014 CrysAlis171 .NET) (compiled May 22 2014,16:03:01) Empirical absorption correction using spherical harmonics, implemented in SCALE3 ABSPACK scaling algorithm.

XRF

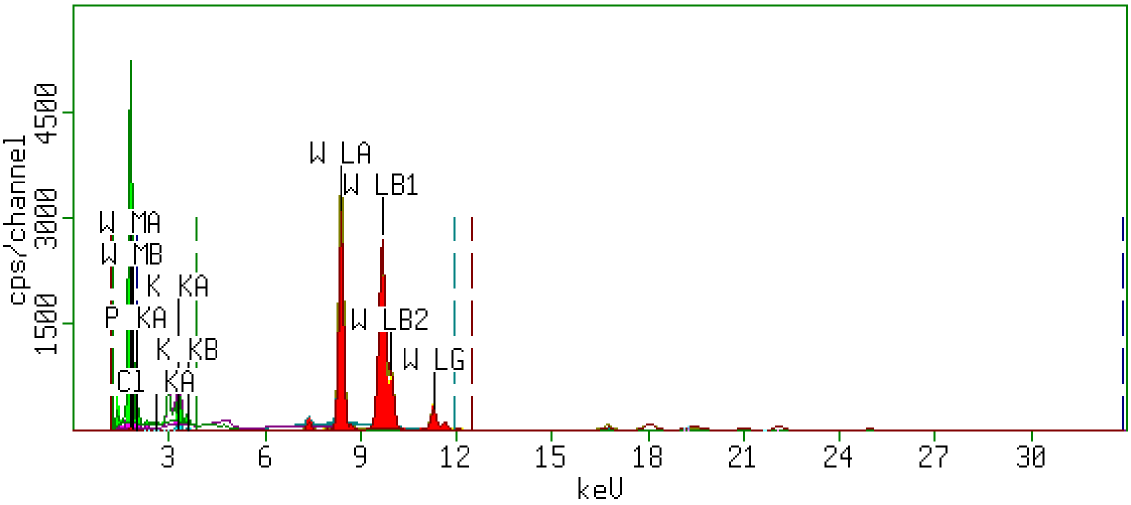

Figure S7. XRF spectrum of **α-WD.**

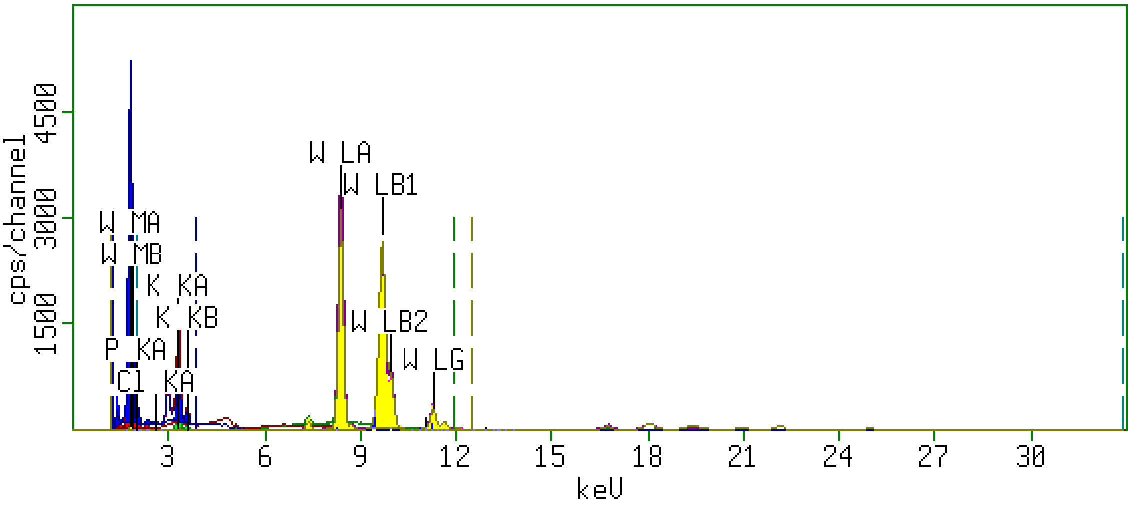

Figure S8. XRF spectrum of **α_2-WD.**

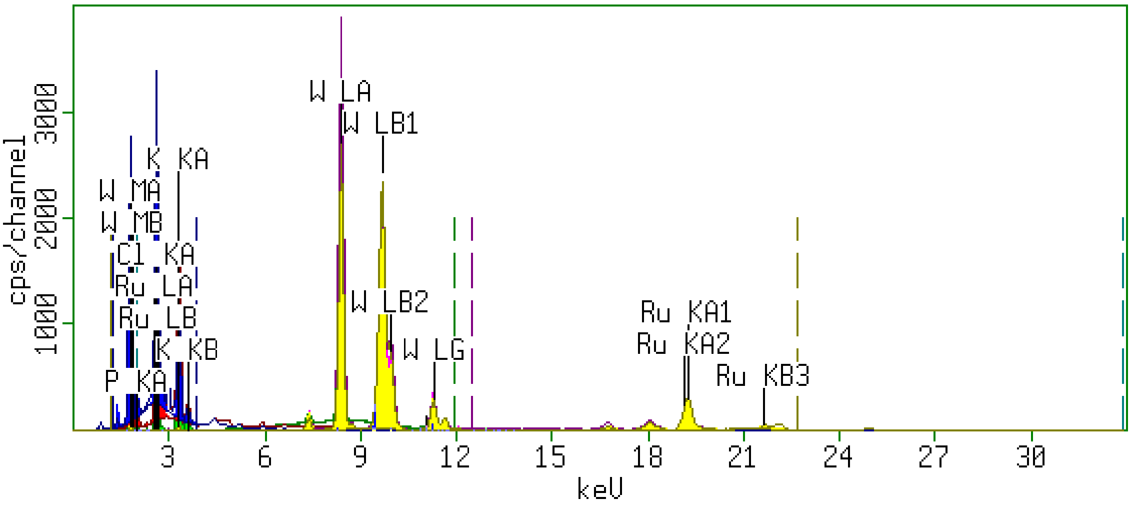

Figure S9. XRF spectrum of **Ru-WD.**

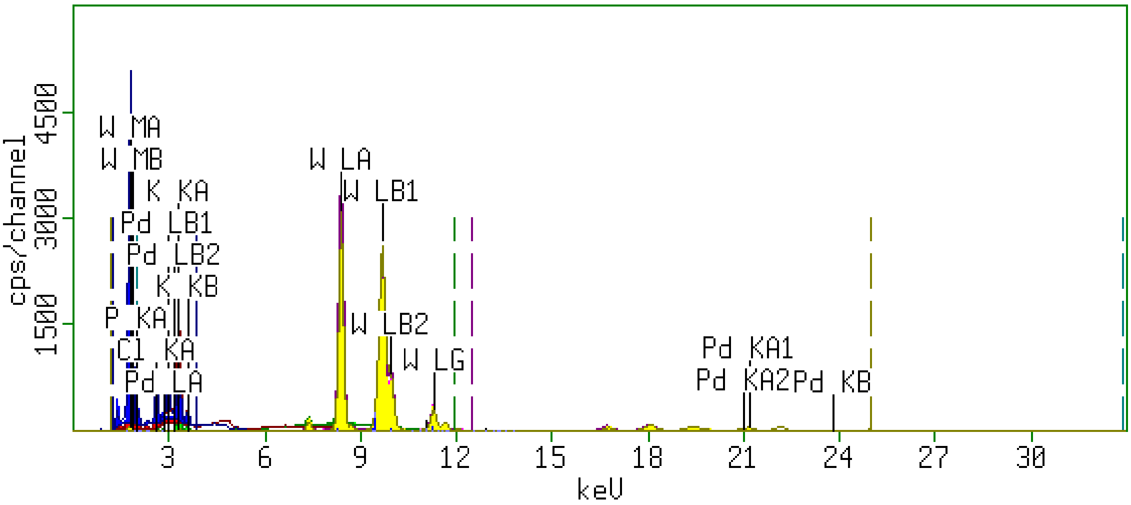

Figure S10. XRF spectrum of **Pd-WD.**

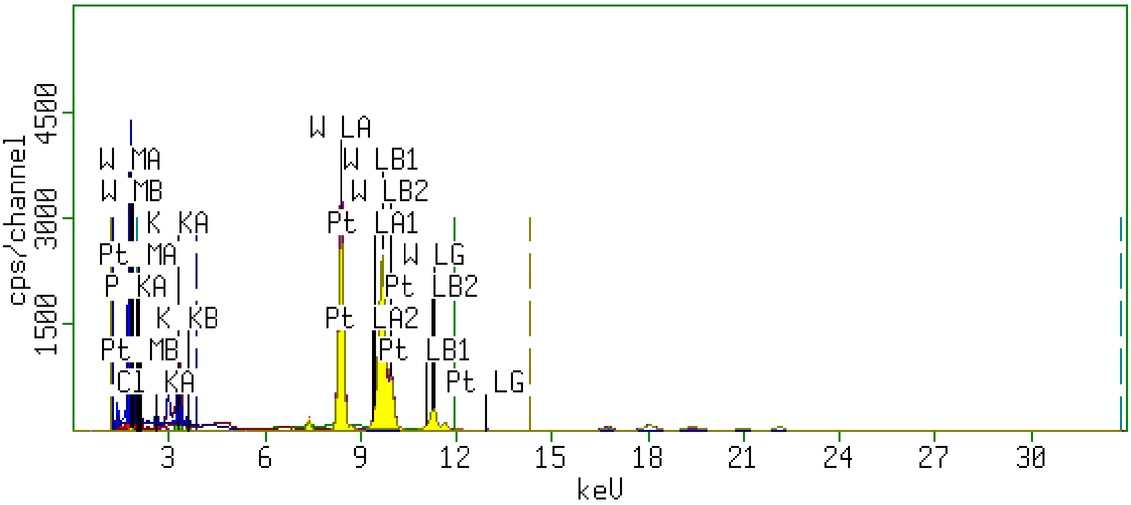

Figure S11. XRF spectrum of **Pt-WD.**

XRF calibration

Table S3. Raw XRD data acquired (counts) and calculated ratios thereof.

POM	XRF Counts			Theoretical Molar Conc.
	P	W	W/P	
α-WD	1501.690	75969.37	50.58900	9.0
α_2-WD	1534.458	71447.88	46.56200	8.5

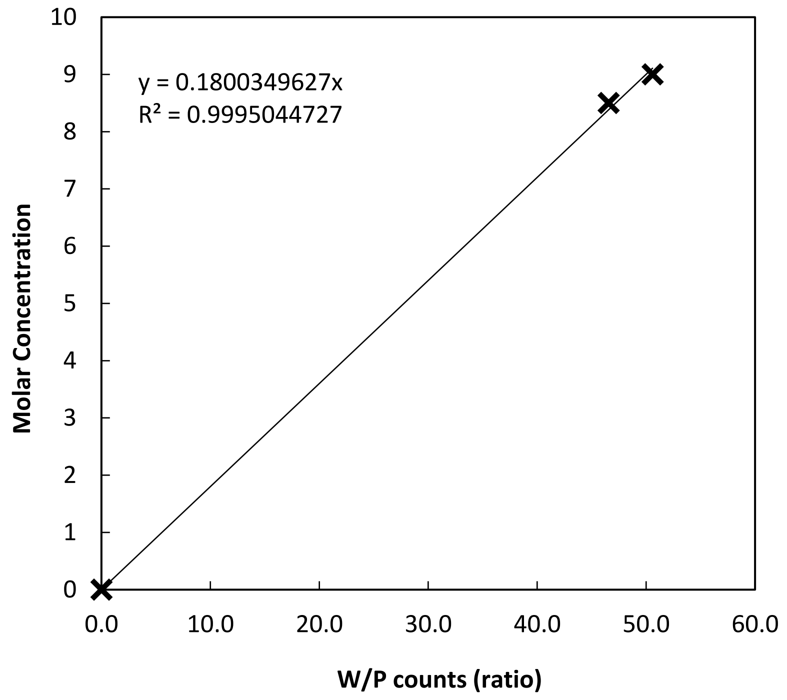

Figure S12. Calibration curve based on data presented in Table S3.

Calculations of Conversion and Selectivities

$$GL\ conv.(\%) = \frac{moles\ (of\ consumed\ C\ in\ GL)}{\text{moles}\ (of\ \ initial\ \ C\ in\ GL)} \times 100 \tag{S1}$$

Equation (S1): Calculation of glycerol conversion.

$$Product\ select.(\%) = \frac{moles\ (C\ in\ product)}{\Sigma\ moles(C\ in\ all\ products)} \times 100 \tag{S2}$$

Equation (S2): Calculation of product selectivity.
